# Azacitidine Is Well-Tolerated and Is Associated with High Response Rate in Elderly Patients with Higher-Risk Myelodysplastic Syndromes: A Single Center Observational Study

**DOI:** 10.3390/cancers18132131

**Published:** 2026-06-30

**Authors:** Nupur Krishnan, David Yanni, Leah Kogan, Lauren Gerard, Jesse McLean, Rouslan Kotchetkov

**Affiliations:** 1 Michael G. DeGroote School of Medicine, McMaster University, Hamilton, ON L8P 1H6, Canada; nupur.krishnan@medportal.ca; 2 School of Medicine, University of Lancashire, Preston PR1 7BH, UK; dyanni@lancashire.ac.uk; 3 School of Medicine, University of Limerick, Castletroy, V94 T9PX Limerick, Ireland; 22183698@studentmail.ul.ie; 4 Hudson Regional Cancer Program, Royal Victoria Regional Health Centre, Barrie, ON L4M 6M2, Canada; gerardl@rvh.on.ca; 5 RVH Research Institute, Royal Victoria Regional Health Centre, Barrie, ON L4M 6M2, Canada; mcleanje@rvh.on.ca

**Keywords:** azacitidine, MDS, elderly, efficacy, safety

## Abstract

Background/Objectives: Azacitidine (AZA) is the standard of care for patients with higher-risk Myelodysplastic Syndromes (MDS). There is limited real-life data, however, characterizing its efficacy and safety profile in elderly patients. Methods: We conducted a single-center retrospective cohort chart review to compare front-line AZA therapy in patients ≥75 years (elderly) vs. <75 years (younger) with higher-risk MDS treated at our cancer center. The primary endpoint was overall survival and main secondary endpoints included response, as per the 2006 International Working Group consensus criteria, leukemia-free survival, transfusion independence, and safety outcomes. Results: In total, 55 patients were elderly (median age: 79.9 years), including 27 patients >80 years, and 41 were younger (median age: 69.4 years). Baseline demographic variables were similar between both groups. The majority of elderly patients (98%) received the full dose of AZA (75 mg/m^2^), compared with 90% of younger patients. The median number of AZA cycles was 8 (range: 2–69) in elderly and 7.75 (range: 1–96) in younger patients. Treatment delays occurred in 36.4% of elderly and 29.3% of younger patients, most commonly due to infection complications in both groups (*p* = 0.076). Disease control rates (complete remission + partial remission + stable disease) were 92.9% in the younger subgroup and 96.4% in the elderly subgroup (*p* = 0.154). Relapse occurred in 48.8% of younger patients and 40.0% of elderly patients. Median overall survival (OS) was 17.3 months for the younger subgroup, 15.7 months for the elderly subgroup (*p* = 0.771), and 11.9 months among patients >80 years (*p* = 0.381). Mortality rates and causes of death were similar between both subgroups. Most common causes of death included disease progression, sepsis, febrile neutropenia, and pneumonia. Conclusions: AZA monotherapy resulted in a high response rate and was well-tolerated in elderly patients with higher-risk MDS. These findings remain consistent in the real-world setting despite potential confounding factors that may contribute to inferior outcomes.

## Simple Summary

Azacitidine (AZA) is a drug used to treat patients with Myelodysplastic Syndromes (MDS). We assessed how effective and safe AZA is in elderly patients (≥75 years) in comparison to younger (<75 years) patients, including patients over 80 years of age with higher-risk MDS. We found that elderly patients were able to receive the same amount of AZA as younger patients. About one-third of the patients in both age groups needed to temporarily stop AZA treatment, primarily due to infections. Both younger and elderly patients had good rates of disease control (including complete remission, partial remission, and stable disease) to AZA therapy and had similar survival times, ranging from 11.9 to 17.7 months. About 60% of patients experienced a significant improvement in their blood cell counts and no longer needed red blood cell transfusions. However, nearly half of the patients eventually experienced a return of their disease. The main causes of death were similar in both age groups and included disease progression and infections. Overall, our results show that, in the real-world setting, AZA is an effective and well-tolerated treatment for elderly patients with higher-risk MDS.

## 1. Introduction

Myelodysplastic syndromes (MDS) are a group of clonal myeloid bone marrow disorders, characterized by abnormal and ineffective hematopoiesis contributing to peripheral blood cytopenias [1,2]. As opposed to non-neoplastic clonal myeloid disorders, MDS is associated with a dysplastic, hypercellular bone marrow [1,2]. Patients with high-risk MDS subtypes have increased potential to develop Acute Myeloid Leukemia (AML), an aggressive malignancy associated with rapid proliferation of blasts and poor prognosis [1,3]. In patients with higher-risk MDS, goals of therapy include improving survival, delaying disease progression, and decreasing disease burden [4]. Treatment options for elderly patients with higher-risk MDS are limited, primarily due to age-related comorbidities, functional impairment, poor tolerability of systemic therapies, patient preferences, and often physician reluctance [5].

Azacitidine (AZA) is a cytidine analogue with a multimodal mechanism of action, inhibiting RNA metabolism, protein synthesis, and DNA methylation [6,7].

As shown in a phase 2 trial, clinical response to azacitidine in MDS is associated with distinct DNA methylation changes in hematopoietic stem and progenitor cells [8]. As a hypomethylating agent, AZA may enable cellular differentiation and re-expression of silenced tumor suppressor genes, and at higher doses can also have direct cytotoxic effects on actively dividing cells [6,9].

AZA has been studied in clinical studies since 1967 [10], and has consistently yielded promising findings in subsequent clinical trials. In 1993, the Cancer and Leukemia Group B (CALBG) carried out the first phase II study assessing the role of AZA in patients with higher-risk MDS, with AZA administered as a continuous intravenous infusion for seven days at a time, every 28 days. This trial demonstrated that AZA treatment resulted in clinically meaningful responses (either complete remission, partial remission, or resolution of all lines of cytopenias) in 49% of evaluable patients [11]. Based on encouraging results of safety and efficacy of azacitidine in MDS, shown in Phase I and II studies [12], the CALBG group published the first randomized trial (CALB 9221) assessing outcomes with AZA. This trial demonstrated once again that in MDS patients, treatment with AZA resulted in significantly higher response rates, improved survival and quality of life, and reduced risk of transformation to AML [13]. Since the release of these pioneering studies, several additional randomized trials have been conducted, including the landmark AZA-001 Phase III, international, multicenter, randomized trial, which confirmed that AZA not only confers a significant survival benefit, but is also well-tolerated, with an acceptable safety data profile [14]. Taken together, these trials provide robust evidence supporting the use of AZA as first-line therapy for higher-risk MDS patients. In clinical practice, AZA is the standard of care in many countries across the world. For example, The Canadian Consortium on Evidence-Based Care in MDS recommends 5-AZA as first-line therapy in all MDS patients with high-intermediate and high-risk IPSS scores, including WHO-defined AML (20–30% blasts) who cannot proceed immediately to allogeneic stem cell transplant [15]. Studies in real-world settings, however, often show inferior outcomes of AZA treatment as compared to RCTs, felt potentially to be due to underutilization and poor persistence with the treatment regimen [16,17].

There is limited evidence assessing the role and safety profile of AZA in higher-risk MDS in the elderly patient population. Santini et al. (2023) conducted a monocentric, pilot phase II study involving 11 elderly patients with higher-risk MDS who were treated with oral AZA, and found that AZA was both a feasible and effective treatment option [18]. Nevertheless, this trial involved only a limited number of patients and, together with post hoc subset analyses from select clinical trials [19], constitutes one of few studies specifically exploring survival outcomes and safety of AZA in elderly patients with higher-risk MDS. Elderly patients, particularly those aged ≥75 years and >80 years, are frequently underrepresented in clinical trials due to frailty, pre-existing comorbidities, or functional limitations. As a result, outcomes observed in clinical trial populations may not be fully generalizable to routine clinical practice or accurately reflect treatment patterns or outcomes in real-world settings, in which populations often include elderly patients. Furthermore, real-world evidence in this patient population remains limited. The objective of this study, therefore, was to characterize the efficacy and safety of monotherapy with AZA in elderly patients with higher-risk MDS in a real-world setting.

## 2. Materials and Methods

### 2.1. Patients

We conducted a retrospective chart review of patients at Hudson Regional Cancer Center who received front-line AZA therapy for high-risk MDS between June 2010 and June 2024. There were no restrictions based on Eastern Cooperative Ontario Group (ECOG) performance status, frailty, or age at the time of AZA initiation. Based on information available on chart review, patients were included if they had a diagnosis of high-risk MDS as per WHO 2016 criteria [20], were 18 years or older, and received at least one complete cycle of AZA. Patients were excluded if they received fewer than one cycle of AZA (*n* = 14), were treated with oral AZA (*n* = 8), or proceeded to allogenic stem cell transplantation (allo-SCT) following AZA therapy (*n* = 12), as these factors were felt to have the potential to substantially influence survival outcomes and confound assessment of AZA efficacy. These exclusion criteria were implemented to create a more homogenous cohort, though we do acknowledge their potential for limiting the generalizability of findings or introduction of selection bias toward patients who were able to continue treatment and did not proceed to potentially curative transplantation.

Baseline demographic, clinical, and laboratory data were collected from the time of diagnosis or earliest available assessment. Data were also extracted pertaining to age at MDS diagnosis and AZA initiation, Revised International Prognostic Scoring System (R-IPSS), AZA administration details, hematological response, survival outcomes, and adverse events. All data were extracted and stored using RedCAP 17.0.6 software. Safety and efficacy of AZA was compared between younger (<75 years) and elderly (≥75 years) patient subgroups.

### 2.2. Safety and Efficacy Assessment

Adverse events (AEs) were defined as any untoward medical occurrence that presented during treatment, but did not necessarily have a causal relationship [21]. AEs were extracted from the electronic medical record (EMR) based on collection at each AZA cycle during routine clinical assessment as reported by patients directly or by health care providers. Details about AEs were also collected from EMR data of patient assessments at the Toxicity Assessment Clinic at our cancer center. AEs were graded and recorded according to Common Terminology Criteria for Adverse Events, Version 5 [22]. Hematological response was extracted from available blood counts (including differential), which were obtained once per cycle or more frequently as clinically indicated. For example, patients with cytopenias were monitored more frequently for transfusion requirements and growth factor support at the discretion of the treating physician.

Responses were categorized as complete remission (CR), partial remission (PR), stable disease (SD), treatment failure, or relapse according to the 2006 International Working Group (IWG) criteria for MDS. Complete remission was defined as normalization of peripheral blood counts and bone marrow blasts < 5%. Partial remission was defined as meeting all criteria for CR, except for persistent bone marrow abnormalities with blasts decreasing by over 50% compared to pre-treatment but still remaining >5%. Stable disease was defined as failure to achieve at least PR, but with no evidence of disease progression for at least 8 weeks. Relapse was defined as any of the following: return to pre-treatment bone marrow blast percentage, decrement of ≥50% from maximum remission/response levels in granulocytes or platelets, reduction in hemoglobin (Hb) concentration by ≥1.5 g/dL, or transfusion dependence. Transfusion independence was defined as the absence of requiring red blood cell transfusions for a minimum of 8 consecutive weeks in patients who were transfusion-dependent at baseline [23,24]. Duration of response was assessed and extracted based on clinical and laboratory evaluations.

For analyses of treatment effectiveness, overall response rate (ORR) was defined as the proportion of patients achieving CR or PR. In addition, a disease control rate (DCR) was calculated and defined as the proportion of patients achieving CR, PR, or SD. Response assessments were determined retrospectively based on review of available bone marrow, laboratory, transfusion, and clinical data available as documented in the medical record.

### 2.3. Statistical Analysis

Data were extracted and analyzed for demographic and disease characteristics, including primary or secondary MDS, cytogenetics, Revised International Prognostic Scoring System (R-IPSS) score, number of comorbidities, and ECOG performance status, as well as AZA dose, schedule, and safety outcomes. For each analysis, age was modeled only as a categorical variable (<75 vs. ≥75, and ≥80 subgroup) and patients were stratified into subgroups based on age (younger subgroup: <75 years; elderly subgroup: ≥75 years).

The highest degree of hematological response was assessed, including achievement of transfusion independence. To evaluate disease control, disease-free survival (DFS) was defined as the time from AZA initiation to disease relapse. Overall Survival (OS) was defined as the time from AZA initiation to death from any cause during the follow-up period.

At the analysis cut-off date (31 December 2025), data for patients with no reported events were censored. Survival curves were estimated using the Kaplan–Meier method, and the log-rank test was applied for comparisons. Hazard ratios (HRs) were calculated using Cox proportional hazards models to determine the effect of AZA on OS, adjusting for pre-specified covariates including age, baseline hemoglobin level, ECOG scores, and cytogenetics, as described previously [5]. In our multivariable Cox proportional hazards analysis, we did not apply changes in hemoglobin and platelets from baseline. The Pearson chi-square test of independence was used to assess the association between age group and treatment response. ECOG scores were categorized as 0–1 versus 2–3 as per clinical judgement based on available data from patient records. Interaction between AZA dose and age was assessed using a likelihood ratio test comparing the two models, with results reported as HRs with corresponding 95% confidence intervals (CIs). Statistical analyses were performed using Prism Software, version 9.1.

## 3. Results

### 3.1. Patient Characteristics

**Figure 1:**
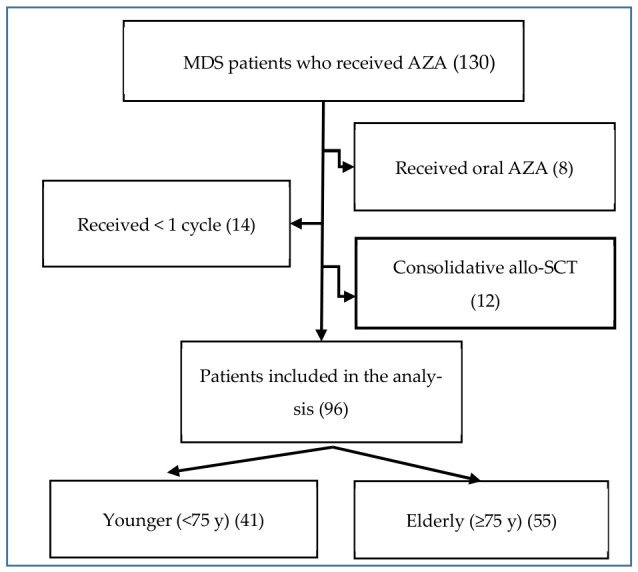
Patient disposition. Abbreviations: MDS, Myelodysplastic Syndromes; AZA, Azacitidine; Allo-SCT, Allogeneic stem cell transplantation.

Between June 2010 and June 2024, 130 patients with MDS received at least one dose of AZA at our cancer center and were screened for eligibility. As per our pre-determined exclusion criteria, patients were excluded if they received less than one full cycle of AZA (*n* = 14), received treatment with the oral AZA formulation (*n* = 8), or proceeded to receive consolidative allo-SCT after AZA therapy (*n* = 12). After applying these criteria, 34 patients were excluded. In total, 96 patients fulfilled the inclusion criteria, consisting of 41 younger patients (<75 years) and 55 elderly patients (≥75 years) (Figure 1). Within the elderly subgroup, 27 patients were over 80 years of age.

Patients’ demographic characteristics are summarized in Table 1.

#### 3.1.1. Demographics and Clinical Characteristics

Among the 96 patients included in the analysis, the median age was 69.4 years (range 54–74) in the younger subgroup, and 79.4 years (range 75–93) in the elderly subgroup (*p* = 0.024). A male predominance was observed in both groups, with ratios of 1.7:1 in younger subgroup and 1.6:1 in the elderly subgroup; however, the difference was not statistically significant. ECOG performance status was similar between groups, with an ECOG score of 3 reported in 10.9% of patients in the elderly group and 12.2% of patients in the younger group (*p* = 0.254). The median number of baseline comorbidities was five in the younger subgroup and six in the elderly subgroup. Secondary malignancies were identified in 46% of younger patients and 29% of elderly patients, with no statistically significant difference (*p* = 0.051).

#### 3.1.2. Disease Characteristics

Disease characteristics were generally comparable between the age groups (Table 2). Primary MDS was observed in 73.2% of younger patients (30/41) and 78.2% of elderly patients (43/55), while secondary MDS was present in 26.8% (11/41) and 21.8% (12/55) of patients, respectively. The median R-IPSS score was 6.5 in the younger subgroup and 5.8 in the elderly subgroup. Poor-risk cytogenetics were more common in the elderly subgroup (18.2%, 10/55) as compared to the younger subgroup (4.9%, 2/41), *p* = 0.013. The proportion of patients with intermediate cytogenetic risk was similar between both subgroups (13/41, 31.7% of younger vs. 17/55, 30.91% of elderly patients), *p* = 0342. The chi2 analysis of all 4 categories of cytogenetics, including missing data, showed chi2 of 4.200, *p* = 0.241. The results of an additional sensitivity chi2 analysis with just those patients who have cytogenetic data available revealed similar chi2 of 2.829 and similarly non-statistically significant *p* value (0.243).

### 3.2. Treatment Administration

Treatment details are summarized in Table 3 below.

In both the younger and elderly subgroups, the majority of patients received the full dose of AZA (75 mg/m^2^), accounting for 90% and 98% of patients, respectively. The median number of AZA cycles administered was 7.75 in the younger subgroup and 8 in the elderly subgroup. Treatment delays occurred in 29.3% of younger patients (*n* = 12), with most common causes including sepsis, febrile neutropenia, injection site reactions, and pneumonia. In the elderly subgroup, 36.5% of patients (*n* = 20) experienced treatment delays, with febrile neutropenia, infections, personal scheduling factors, and bowel obstruction being the most frequently reported causes. The difference in treatment delays between the two subgroups was not statistically significant (*p* = 0.076).

### 3.3. Efficacy Assessment

**Figure 2:**
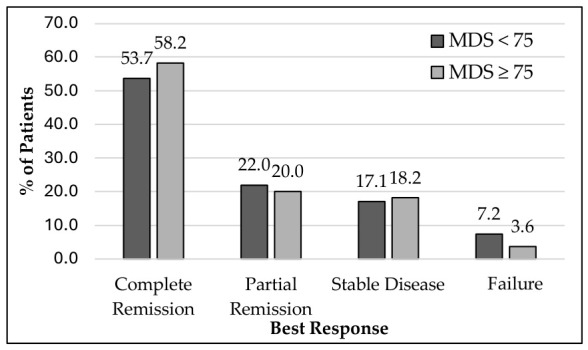
Response rates, represented in percentages, (%) to Azacitidine therapy in patients <75 and ≥75 years of age.

Response rates and depth of response were comparable between the two subgroups, as shown in Figure 2.

**Figure 3:**
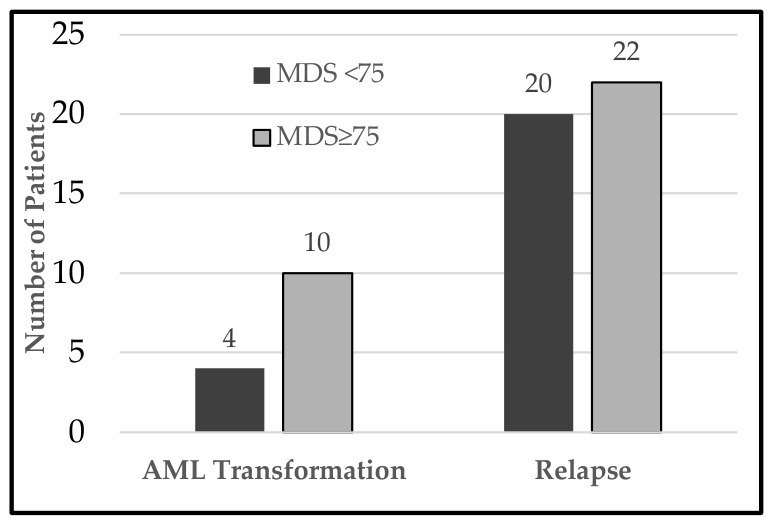
Rate of transformation to acute myeloid leukemia and relapse after initial response. Results are presented as number of patients. Abbreviations: AML, Acute Myeloid Leukemia.

Disease control rate (including complete remission, partial remission, and stable disease) was achieved in 92.8% of patients in the younger subgroup and 96.4% in the elderly subgroup (*p* = 0.154). The Pearson chi-square test of independence was used to assess the association between age group and treatment response. Among 96 patients, response to therapy did not differ significantly by age group (<75 vs. ≥75 years). In patients aged <75 years (*n* = 41), 53.7% achieved complete remission, 22.0% partial remission, 17.1% stable disease, and 7.3% had progressive disease. Among patients aged ≥75 years (*n* = 55), corresponding proportions were 58.2%, 20.0%, 18.2%, and 3.6%, respectively. There was no statistically significant association between age group and response (Pearson χ^2^ = 0.76, *p* = 0.86). Disease relapse, after reaching remission, occurred in 48.8% of younger patients (*n* = 20) and 40.0% (*n* = 22) of elderly patients. Transformation to AML was more frequent in the elderly subgroup, occurring in 18.18% of patients (*n* = 10) compared to 9.76% (*n* = 4) in the younger subgroup (*p* < 0.016) (Figure 3).

**Figure 4:**
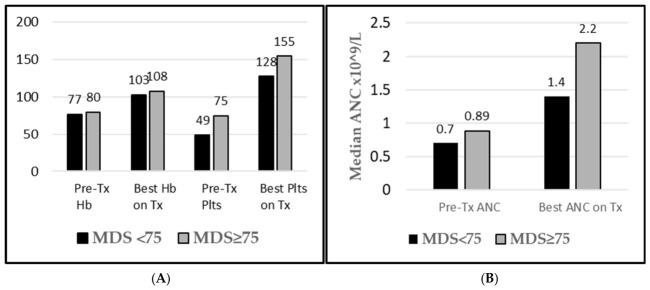
Hematological response to azacitidine therapy. (**A**) Change in median hemoglobin (g/L) and platelet count (×10^9^/L) from baseline to the best-achieved value. (**B**) Change in median absolute neutrophil count (×10^9^/L) from baseline to the best-achieved value. Abbreviations: ANC, Absolute neutrophil count; plt, platelet count; Hb, Hemoglobin; Tx, treatment.

Hematologic responses were observed in both the younger and elderly subgroups as shown in Figure 4 below.

In the younger subgroup, the median hemoglobin at baseline was 77 g/L (range: 46–100), increasing to a median best value of 103 g/L (range: 74–152) with AZA treatment. In the elderly subgroup, median hemoglobin improved from 80 g/L (range: 56–140) at baseline to 108 g/L (range: 70–155) (Figure 4A). In the younger subgroup, the median ANC at baseline was 0.70 × 10^9^/L (range: 0–27 × 10^9^/L), increasing to a median best ANC of 1.4 × 10^9^/L (range: 0.02–12.3 × 10^9^/L) with AZA treatment. In the elderly subgroup, the median baseline ANC was 0.89 × 10^9^/L (range: 0.02–58 × 10^9^/L), which improved to 2.2 × 10^9^/L (range: 0.04–9.6 × 10^9^/L) with treatment (Figure 4B). Platelet counts also improved in both groups. In the younger subgroup, the median platelet count increased from 49 × 10^9^/L at baseline (range: 2–600 × 10^9^/L) to a best achieved platelet count of 128 × 10^9^/L (range 21–474 × 10^9^/L). In the elderly subgroup, median platelet counts rose from 75 × 10^9^/L (range: 16–558 × 10^9^/L) to 155 × 10^9^/L (range: 10–767 × 10^9^/L) on AZA (Figure 4A).

**Figure 5:**
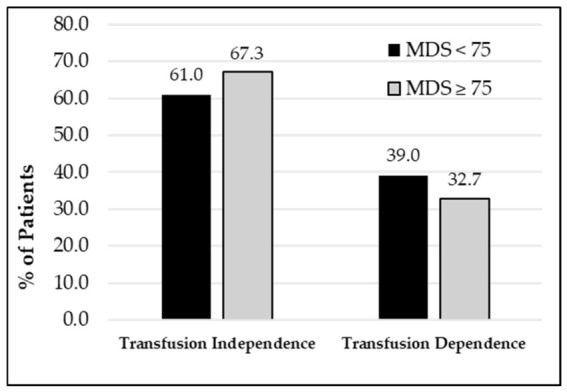
Rate of patients, presented as percentage (%) of patients, who reached transfusion independence and those who remained on supportive red blood cell transfusions at their best response time.

Red blood cell transfusion independence was achieved by 61.0% (*n* = 25) of younger patients and 67.3% (*n* = 37) of elderly patients, with no statistically significant difference between groups (*p* = 0.156) (Figure 5).

**Figure 6:**
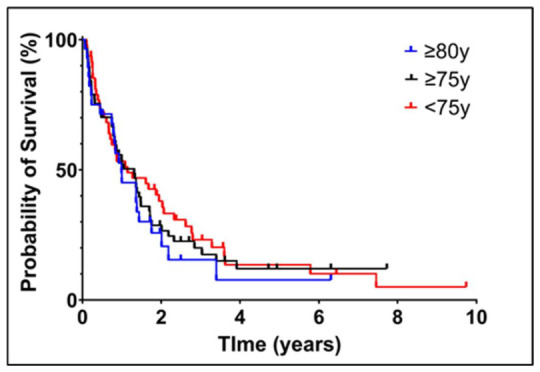
Overall survival for younger subgroup (<75 years), elderly subgroup (≥75 years), and patients ≥80 years of age.

Median OS for younger, elderly, and patients ≥80 years of age is shown in Figure 6. OS in the younger subgroup was 17.3 months, compared to 15.7 months in the elderly subgroup (HR 0.91, 95% CI 0.59–1.41, *p* = 0.771). For patients ≥80 years, OS was 11.9 months, with no statistically significant difference compared to the rest of the elderly patients (*p* = 0.381) (Figure 6).

Leukemia free survival (LFS) was 14.1 months in younger patients and 12.1 months in elderly patients (HR 0.91, 95% CI 0.59–1.39, *p* = 0.739). In patients over 80 years, LFS was 9.5 months.

Ninety six patients were included in the multivariable Cox proportional hazards analysis. There were 93 events in the multivariable Cox proportional hazards analysis. Age group (<75 vs. ≥75 years) was not significantly associated with overall survival (HR 1.78, 95% CI 0.71–4.44; *p* = 0.218). No significant associations with overall survival were observed for baseline cytogenetic risk (HR 1.15, 95% CI 0.85–1.56; *p* = 0.375), ECOG performance status (HR 1.26, 95% CI 0.90–1.77; *p* = 0.183), baseline hemoglobin (HR 0.99, 95% CI 0.96–1.01; *p* = 0.278), baseline absolute neutrophil count (HR 1.02, 95% CI 0.99–1.06; *p* = 0.233), or baseline platelet count (HR 1.00, 95% CI 1.00–1.00; *p* = 0.443). The overall model was not statistically significant (likelihood ratio χ^2^ = 13.13, *p* = 0.157).

### 3.4. Safety

Treatment-emergent adverse events are summarized in Table 4. In the elderly subgroup, neutropenia occurred in 43.6% of patients (*n* = 24), including 2 patients (3.6%) with Grade 3 neutropenia and 1 patient (1.8%) with Grade 4 neutropenia. Thrombocytopenia occurred in 25.4% of elderly patients (*n* = 14), including 2 patients (3.6%) who developed Grade 3 thrombocytopenia. No elderly patients developed Grade 4 thrombocytopenia. Anemia occurred in 34.5% of elderly patients (*n* = 19), including 3 patients (5.4%) with Grade 3 anemia and 2 patients (3.6%) who developed Grade 4 anemia.

In the younger subgroup, neutropenia occurred in 29.3% of patients, including 1 patient (2.4%) who had Grade 3 neutropenia and 3 patients (7.3%) with Grade 4 neutropenia. Thrombocytopenia occurred in 29.3% of younger patients, including 3 patients (7.3%) who developed Grade 3 thrombocytopenia. No Grade 4 thrombocytopenia was observed in the younger group. Anemia occurred in 26.8% of younger patients (*n* = 11), including 4 patients (9.8%) who developed Grade 3 anemia and 1 patient (2.4%) who developed Grade 4 anemia. All events were recorded during treatment with AZA. There were no statistically significant differences between the younger and elderly groups.

**Figure 7:**
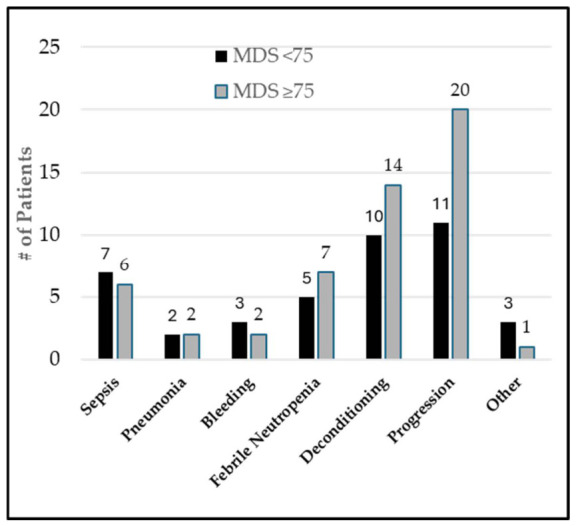
Causes of death in younger and elderly MDS patients treated with AZA.

A total of 41 patients in the younger subgroup and 52 patients in the elderly subgroup died. The causes of death are shown in Figure 7.

Disease progression was the most common cause of death in both groups, and occurred more frequently in the elderly subgroup (36.4%, *n* = 20) as compared with the younger subgroup (26.8%, *n* = 11), although this difference did not reach statistical significance (*p* = 0.078). Deaths due to febrile neutropenia were similar between subgroups (12.2% in younger subgroup vs. 12.7% in elderly subgroup). Bleeding-related mortality was more frequent in the younger subgroup (7.3% vs. 3.6%; *p* = 0.041).

## 4. Discussion

Our study showed that even in elderly patients with higher-risk MDS, AZA continues to be effective and well-tolerated in a community setting. These results are particularly notable, as they reflect real-world clinical practice, where patients often present with confounding factors, such as poorer baseline ECOG status and more comorbidities that could otherwise contribute to inferior outcomes and cannot be as tightly controlled for as in clinical trials.

The efficacy and safety of AZA have been well-established in clinical trials. AZA-001, a Phase III, international, multicenter, randomized prospective trial, demonstrated a significant improvement in OS with AZA treatment in patients with higher-risk MDS [14]. In the AZA-001 trial, patients received a median of 9 cycles of AZA, comparable to the treatment exposure observed in both our elderly and younger subgroups (median: 8 and 7.75 cycles, respectively). The median OS in AZA-001 was 24.4 months, exceeding that observed in both our elderly (15.7 months) and younger (17.3 months) subgroups. Overall, while our elderly and younger subgroups demonstrated shorter OS compared to the AZA-001 results, they exhibited comparable treatment durations [14]. We did not find any new patterns of hematological adverse events in either group, and no new non-hematological adverse events were identified. In addition, no age-related pattern of AEs were observed, including AML transformation or death due to hemorrhage.

These broadly similar outcomes were observed despite several key differences between the AZA-001 patient population and our cohorts. Notably, the average age of the AZA-001 sample (69 years) was comparable to the median of our younger subgroup (69.4 years) but substantially lower than our elderly subgroup (79.4 years). Furthermore, eligibility for AZA-001 required an ECOG status of 0–2 and an estimated life expectancy of at least three months. In contrast, 10.9% of patients in our elderly subgroup and 12.2% in our younger subgroup had an ECOG of 3, and both subgroups had a median of 5–6 comorbidities at baseline. Furthermore, although treatment delays were permitted and did occur in AZA-001 as needed to allow for blood count recovery, they were not otherwise influenced by non-medical factors. In our real-world cohort, however, patient-related personal scheduling factors were among the leading reasons for treatment delays. Despite these differences in age, baseline performance, and real-world treatment constraining factors, our findings suggest that the tolerability and safety profile of AZA is similar in the real-world setting as compared to clinical trial environments.

Several real-world studies have evaluated the safety and efficacy of AZA in patients with higher-risk MDS. One of the earliest was conducted by Bernal and colleagues (2015), comparing OS in high-risk MDS patients treated with AZA versus conventional treatment, using data from the Spanish registry over the course of a decade [25]. This real-world study demonstrated a median OS with AZA of 13.4 months. This finding was shorter than that reported in the AZA-001 clinical trial as well as both our elderly and younger subgroups. Moreover, patients in the Bernal et al. cohort received a median of six cycles of AZA; fewer than in AZA-001 (9 cycles) and both our elderly and younger subgroup (8 and 7.75 cycles respectively). The average age of the study population was 74 years, which, although higher than that of the AZA-001 sample, remained lower than the median age of our elderly subgroup. Even so, the authors still suggested that this advanced age, in addition to presence of comorbidities and variations in clinical practice, may have contributed to the inferior outcomes observed in their real-world setting as compared to those of AZA-001 [25]. Despite these considerations, given that our findings include improved OS and treatment tolerability despite a higher median age, our results suggest that even in populations characterized by more advanced age and a substantial burden of comorbidities, AZA continues to demonstrate meaningful efficacy and an acceptable safety profile.

Another real-world study, by Helbig and colleagues (2017), similarly demonstrated that AZA is more effective than conventional care in patients with higher-risk MDS. Despite a younger median patient age (67 years) compared with even our younger subgroup, this study reported a comparable median number of six AZA cycles, but a lower complete remission rate of 14%, compared to 53.7% and 58.2% in our elderly and younger subgroups, respectively [26]. Consistent findings were reported in a large-scale real-world study by Mozessohn et al., (2018) which also observed a median of six AZA cycles, a CR rate of 17%, and an OS of 11.6 months [27]. Together, despite having younger patient populations and similar treatment exposure, these two studies observed lower response rates as compared to our cohort of elderly patients with higher-risk MDS. These findings are also consistent with those reported by Wiśniewski and colleagues (2024), who evaluated AZA treatment in patients with MDS in a real-world setting, and observed a CR rate of 13.9%, median OS of 17.6 months, and median PFS of 14.96 months [28]. Similarly, a real-world, retrospective study by Rajakumaraswamy and colleagues (2024), evaluating effectiveness of AZA in treatment-naive patients with higher-risk MDS reported a similar OS (17.9 months) to that identified in the cohort studied by Wiśniewski and colleagues, but even lower rates of CR of 7.9% [29]. Notably, this cohort had a median age of 74 years and only 0.5% of patients had an ECOG status greater than 2 [29].

Although these studies have explored the efficacy and safety profile of AZA in the real-world setting, few have specifically focused on outcomes in elderly patient populations. Taken together, however, they do not demonstrate a consistent association between advanced age and lower CR or shorter OS. In fact, compared to these previous studies, data from both of our patient subgroups suggest higher CR rates and similar or longer OS, irrespective of median age [26,27,28,29]. These findings once again suggest that AZA remains an effective and safe treatment option for elderly patients with higher-risk MDS while further highlighting the need to identify prognostic factors beyond chronological age alone. When interpreting the results of our study, however, we do recognize that differences in response definitions—for example DCR vs. ORR—along with the retrospective nature of response assessment, and patient eligibility criteria, may contribute to the relatively high response rates observed in our study as compared to other real-world cohorts.

Several prognostic-scoring systems have been developed, including the International Prognostic Scoring System (IPSS) and the Revised-IPSS (IPSS-R), which estimate prognosis based on bone marrow blast percentage, cytogenetic abnormalities, and peripheral blood counts [2]. Based on these variables, the IPSS-R stratifies patients with MDS into five risk categories: very low, low, intermediate, high, and very high [30]. Additional analyses have sought to further elucidate prognostic factors associated with response rates and survival outcomes in higher-risk MDS patients treated with AZA. In the study by Wiśniewski and colleagues (2024), univariate analysis identified only unfavorable cytogenetic risk as a prognostic factor of lower response rates. A multivariate model, however, demonstrated that older age, higher IPSS risk, and higher IPSS-R cytogenetic risk were all potential independent predictors of shorter OS and, along with serum ferritin levels, shorter PFS [28]. Multivariate analyses by Bernal and colleagues (2015) have actually identified age, as well as IPSS and lactate dehydrogenase levels, but not use of azacitidine, as being predictor variables of OS [25].

Despite these reports linking advanced age to poorer outcomes, patients in our real-world setting—who, on average were older, had higher IPSS-R scores, and overall poorer baseline performance statuses—still demonstrated improved survival with AZA treatment. As data extraction was limited by variables available in patients’ charts, molecular markers such as serum ferritin and lactate dehydrogenase (LDH) levels could not be consistently captured. Based on data that was available, however, in our study, neither baseline characteristics nor markers of hematological recovery were significantly associated with survival. This may be explained by the pattern of AZA administration at our cancer center: we approach AZA as “supportive therapy” targeting sick bone marrow rather than “a chemotherapy drug”. As such, our center minimizes dose reductions due to cytopenias and instead delivers the full dose of AZA while maximizing supportive therapy. This approach, even in elderly patients, may also explain the higher response rate observed in our cohorts as compared to other real-world studies. Moving forward, further research should focus on identifying additional clinical biomarkers that may better predict survival and prognosis, aside from age and baseline performance status alone, particularly given that our findings suggest that AZA may still confer survival benefit despite these factors.

The primary strength of this study lies in the gap it addresses in the available literature. To our knowledge, this is the first study to specifically evaluate the safety and efficacy of AZA in elderly patients with higher-risk MDS in the real-world setting. By utilizing real-world clinical data, this analysis more accurately reflects actual practice patterns and patient outcomes, and is therefore more generalizable to real-world patient populations, as compared to data derived from clinical trials alone.

Limitations of this study are primarily attributable to its retrospective design. Due to the observational nature of this study, causal relationships cannot be established. Furthermore, factors such as baseline patient demographics or unanticipated events interfering with treatment dosing schedules could not be controlled for in the real-world setting and may have therefore introduced confounding factors. In addition, as this was a retrospective chart review, the availability and accuracy of data was dependent on the quality of documentation in the electronic medical record, which may have varied between providers. Key variables, including cytogenetic data and molecular markers, were at times missing or not uniformly reported, which may introduce heterogeneity in outcome reporting and subsequent data extraction. Next, cytogenetic information remains unavailable in a substantial proportion of patients in both age groups. To assess the importance of missing cytogenetics data and its influence on OS, AML transformation, and response outcomes we performed an additional sensitivity analysis with patients who had available cytogenetic information. We found that the missing data does not appear to change the distribution of the cytogenetic categories between <75 and >75 groups, suggesting that the missing data are missing at random, and should not affect subsequent interpretation of OS, AML transformation and other outcomes.

Furthermore, the study’s retrospective design along with the implemented eligibility criteria may have introduced selection bias. Specifically, excluding patients who underwent allogenic stem cell transplantation, were treated with oral AZA, or received less than one full cycle of AZA may limit the generalizability of our findings to patients who were fitter and able to continue treatment, as well as those who did not proceed to potentially curative transplantation. In addition, given the relatively small sample size, the analysis of our multivariable Cox models is exploratory and may be underpowered. Given the retrospective design, small sample size, missing data, and potential selection bias, the results of our study should be interpreted with caution, when applied to different patient populations.

## 5. Conclusions

In conclusion, this retrospective chart review is, to our knowledge, the first to assess the safety and efficacy of AZA in elderly patients with higher-risk MDS in a real-world setting. Compared with results from RCTs, patients in the real-world setting often have shorter survival, higher ECOG status, and a greater burden of comorbidities, all of which may contribute to inferior outcomes and underscore the importance of real-life evidence. Despite these factors, however, our data suggest that in elderly patients with higher-risk MDS, AZA maintains both a high response rate, including complete remission, and an acceptable safety profile. Moving forward, multicenter real-world studies would be valuable to further strengthen the robustness and generalizability of these findings. In addition, future research should aim to identify molecular biomarkers and clinical predictors of response or prognosis.

## Figures and Tables

**Table 1:** Patient Demographics.

	MDS < 75 Years *n* = 41 (42.7%)	MDS ≥ 75 Years *n* = 55 (57.3%)	*p*-Value
Age at Diagnosis, years			
Median	69.4	79.9	0.024 *
Range, Min–Max	54.4–74.5	75–92.6	
Gender			
Male	26 (63.4%)	34 (61.8%)	0.144
Female	15 (36.6%)	21 (38.2%)	
# of Comorbidities			
Median	5	6	0.899
Range, Min–Max	0–18	0–22	
Baseline ECOG			
0	11 (26.8%)	19 (34.5%)	0.093
1	13 (31.7%)	16 (29.1%)	0.077
2	12 (29.3%)	14 (25.5%)	0.572
3	5 (12.2%)	6 (10.9%)	0.254
2° Malignancy	19 (46.3%)	16 (29.1%)	0.051

Results presented as patient number, (%). * Statistically significant. Abbreviations: MDS, Myelodysplastic syndromes; ECOG, Eastern Cooperative Oncology Group.

**Table 2:** Cytogenetics, risk score and other MDS features.

	MDS < 75 Years	MDS ≥ 75 Years	*p*-Value
Cytogenetics			
0: Not available	20 (48.8%)	20 (36.3%)	0.098
1: Good	6 (14.6%)	8 (14.6%)	0.231
2: Intermediate	13 (31.7%)	17 (30.9%)	0.342
3: Poor	2 (4.9%)	10 (18.2%)	0.013 *
MDS			
Primary	30 (73.2%)	43 (78.2%)	0.729
Secondary	11 (26.8%)	12 (21.8%)	0.155
IPSS			
Median	2	2	1.000
Range, Min–Max	0–4	0–4	
R-IPSS			
Median	6.5	5.8	0.989
Range, Min–Max	0–9.5	0–9.5	

Results presented as patient number, (%). * Statistically significant. Abbreviations: MDS, Myelodysplastic syndromes; IPSS, International Prognostic Scoring System; R-IPSS, Revised International Prognostic Scoring System.

**Table 3:** Treatment Details.

	MDS < 75 Years	MDS ≥ 75 Years	*p*-Value
Absolute AZA Dose, mg			
Median	150	140	0.089
Range (Min–Max)	60–180	100–170	
% patients on full dose	90%	98%	0.655
Time on AZA, months			
Median	7.13	9	0.335
Range (Min–Max)	1–96	2–70	
# AZA Cycles			
Median	7.75	8	0.533
Range	1–96	2–69	
Delay	12 (29.3%)	20 (36.5%)	
Duration			
Median, days	17.5	14	0.076
Range, days	7–56	2–120	
Causes:	Sepsis, febrile neutropenia, injection site reaction, pneumonia	Neutropenia, scheduling, infection, bowel obstruction	

Results presented as *n*, (%). Abbreviations: MDS, Myelodysplastic syndromes; AZA, Azacitidine.

**Table 4:** Summary of treatment-emergent adverse events.

	<75 (*n* = 41)		≥75 (*n* = 55)	
	*n*	%	*n*	%
Total hematological AEs	15	36.59	29	52.73
Total Neutropenia	12	29.27	24	43.64
Grade 3 Neutropenia			2	3.6
Grade 4 Neutropenia			1	1.8
Total Thrombocytopenia	12	29.27	14	25.45
Grade 3 Thrombocytopenia	3	7.3	2	3.6
Grade 4 Thrombocytopenia	0	0	0	0
Total Anemia	11	26.83	19	34.55
Grade 3 Anemia	4	9.8	3	5.4
Grade 4 Anemia	1	2.4	2	3.6
Febrile Neutropenia	4	9.76	14	25.45
Clinical Bleeding	1	2.44	1	1.82
Peripheral Bruising	1	2.44	1	1.82
Pneumonia	5	12.20	6	10.91
Injection Site Infection	3	7.32	7	12.73
Sepsis	3	7.32	1	1.82
Nausea	8	19.51	11	20.00
Diarrhea	4	9.76	6	10.91
Constipation	6	14.63	10	18.18
Fatigue	3	7.32	8	14.55

Results presented as *n*, %. Abbreviations: AEs = Adverse events.

## Data Availability

Data is unavailable due to privacy or ethical restrictions.

## Abbreviations

The following abbreviations are used in this manuscript:

AE	Adverse events
AZA	Azacitidine
Allo-SCT	Allogeneic stem cell transplantation
AML	Acute Myeloid Leukemia
ANC	Absolute neutrophil count
CALBG	Cancer and Leukemia Group B
CI	Confidence interval
CR	Complete remission
DNA	Deoxyribonucleic acid
ECOG	Eastern Cooperative Oncology Group
Hb	Hemoglobin
HR	Hazard ratio
LFS	Leukemia free survival
MDS	Myelodysplastic Syndromes
OS	Overall survival
PR	Partial remission
R-IPSS	Revised International Prognostic Scoring System
RNA	Ribonucleic Acid
SD	Stable disease
WHO	World Health Organization
